# A well-preserved aneuretopsychid from the Jehol Biota of China (Insecta, Mecoptera, Aneuretopsychidae)

**DOI:** 10.3897/zookeys.129.1282

**Published:** 2011-09-16

**Authors:** Dong Ren, ChungKun Shih, Conrad C. Labandeira

**Affiliations:** 1 College of Life Sciences, Capital Normal University, Beijing 100048, China; 2 Department of Paleobiology, Smithsonian Institution, National Museum of Natural History, Washington, DC 20013 USA; 3 Department of Entomology and BEES Program, University of Maryland, College Park, MD 20742 USA

**Keywords:** Aneuretopsychidae, new genus, new species, proboscis, Yixian Formation, China, Early Cretaceous, pollination drops

## Abstract

The Aneuretopsychidae is an unspeciose and enigmatic family of long-proboscid insects that presently consist of one known genus and three species from the Late Jurassic to Early Cretaceous of north-central Asia. In this paper, a new genus and species of fossil aneuretopsychid is described and illustrated, *Jeholopsyche liaoningensis* **gen. et sp. n.** Fossils representing this new taxon were collected from mid Early Cretaceous strata of the well known Jehol Biota in Liaoning Province, China. This finding documents the first formal record of fossil Aneuretopsychidae in China. In addition, this well-preserved and new material reveals previously unknown and detailed morphological structure of the mouthparts, antennae, head, thorax, legs and abdomen of this distinctive insect lineage.

## Introduction

The Aneuretopsychidae is a depauperate and enigmatic, extinct family of mid Mesozoic Mecoptera erected by Rasnitsyn and Kozlov (1990). Currently, only one genus with three species has been described. *Aneuretopsyche rostrata* and *Aneuretopsyche minima* were found in the Upper Jurassic Karabastau Formation of the South Kazakhstan (formerly Chimkent) Province in Kazakhstan, and *Aneuretopsyche vitimensis* was recovered from the Lower Cretaceous Zaza Formation along the upper reaches of Vitim River, at the Baisa locality of western Transbaikalia in Russia (Rasnitsyn and Kozlov 1990). The establishment of the Aneuretopsychidae was very significant, as it was the first time that a mecopteroid insect was found to have exceptionally prolonged, siphonate mouthparts. The Aneuretopsychidae was initially and tentatively placed by Rasnitsyn and Kozlov (1990) into the Suborder Aneuretopsychina within the scorpionfly clade (Order Panorpida = Mecoptera). Moreover, the taxa was thought to have interesting feeding adaptations similar to cicadas and butterflies, particularly as it was difficult to interpret the first, enigmatic, proboscis observed in Mecoptera of any age.

The Aneuretopsychidae, together with mid Mesozoic Mesopsychidae (Ren et al. 2010a), and Pseudopolycentropodidae (Ren et al. 2010b), and now the Late Permian to Early Triassic Nedubroviidae (Bashkuev 2011), form a particularly interesting clade, the Aneuretopsychina, which lasted 150 million years from the Late Permian to the latest Early Cretaceous. This essentially preangiospermous lineage exhibited a tubular siphonate proboscis, indicating that mid Mesozoic scorpionflies were accessing pollination drops from gymnospermous plants, discussed elsewhere (Labandeira et al. 2007; Ren et al. 2009; Labandeira 2010). Such a mouthpart structure was convergent with and homologous to labial mouthpart elements of the brachycerous dipteran proboscis, but analogous to the maxillary glossae of lepidopterans which evolved during the Early Cretaceous (Labandeira et al. 2007; Labandeira 2010). Grimaldi et al. considered that these three families were not true mecopterans but rather stem-group mecopteroids showing remarkable parallel evolution with the Diptera (Grimaldi and Engel 2005; Grimaldi et al. 2005). By contrast, Ren and colleagues (Ren et al. 2009) indicated that this clade represents a major, early-derived lineage of basal Mecoptera.

Recently, we collected two fossils of Aneuretopsychidae, the first an excellently preserved part and counterpart, and a second poorly-preserved specimen lacking a counterpart, both from the well-known Yixian Formation. The Jehol Biota of the Yixian Formation has yielded abundant fossil insects associated with seed plants, some of which were potential pollen or nectar-feeders, such as members of the Orthoptera, Heteroptera, Coleoptera, Hymenoptera and Diptera (Ren 1998; Ren et al. 2010c). The new taxon reported here provides new morphological details for evaluating the phylogenetic position of this interesting family. Based on its unique venational characters, we erect a new genus with a new species, *Jeholopsyche liaoningensis* gen. et sp. n. The binomial name *Jeholopsyche liaoningensis* has appeared for the first time in Ren et al. (2009). However, diagnostic characters of this genus were mentioned only in the electronic Supporting Online Material, and were therefore not effectively published in terms of the International Code of Zoological Nomenclature (Article 9; ICZN 1999). We republish this genus and species herein by providing the required detailed description and other information to ensure no conflict with the Code and make the names nomenclatorially available from this paper.

The Early Cretaceous specimens were collected from the Yixian Formation at Huangbanjigou Village, in Shangyuan Township, near Beipiao City of Liaoning Province, China. There is general agreement that the age of the Yixian Formation is of late Barremian age, based on 40Ar/39Ar date of 125 Ma on sanidine and biotite minerals (Swisher et al. 2002), confirmed by 235U/207Pb dates on zircon crystals (Wang et al. 2001). This is the date that we accept herein. However, this date remains contentious, particularly for some Chinese scientists. Three potential ages historically have been proposed for the Yixian fossils; namely, the Late Jurassic (Ren et al. 1997; Zheng et al. 2003), the transition from the Late Jurassic to the Early Cretaceous (Chen et al. 2004; Wang et al. 2004; Wang et al. 2005), and the Early Cretaceous (Swisher et al. 1999; Zhou et al. 2003).

## Materials and methods

This study is based on two fossil specimens housed in the fossil insect collection of the Key Laboratory of Insect Evolution & Environmental Changes, College of Life Sciences, Capital Normal University, Beijing, China (CNUB; Dong Ren, Curator). The specimens were examined using a Leica MZ12.5 dissecting microscope, and illustrated with the aid of a drawing tube and formatted through Adobe Photoshop CS2 software.

The wing venation nomenclature used in this paper is based on the interpretations and system proposed by Novokshonov (Novokshonov 2002).

## Systematic palaeontology

### Family Aneuretopsychidae

Rasnitsyn & Kozlov, 1990

http://species-id.net/wiki/Aneuretopsychidae

#### Type genus: 

*Aneuretopsyche* Rasnitsyn & Kozlov, 1990

#### Emended diagnosis.

 Moderate-sized insects, mouthpart position most probably opisthognathous; hypognathous placement possible. Adults have a remarkably prolonged, siphonate proboscis, its exterior covered with well-developed dense hairs or microtrichia arranged in distinct annulae; terminus surrounded by a distinctive, lobed, fleshy pseudolabellum. Antennae distinctly longer than proboscis, multiarticulate; articles covered with annulate hairs. Forewing elongate. Sc with multiple branches. Both Rs and MA bifurcating; MP 4-branched. CuA single or probably bifurcating. Hindwing distinctly broader than forewing. Hairs on legs arranged in distinctive rings.

#### Included genera.

 In addition to the type genus, *Jeholopsyche* gen. n.

### Genus Jeholopsyche gen. n.

urn:lsid:zoobank.org:act:EB76FD90-EF08-4907-B75A-71FF4853D614

http://species-id.net/wiki/Jeholopsyche

#### Type species:

*Jeholopsyche liaoningensis* sp. n.

#### Etymology.

 The genus name is derived from the Jehol Biota; and *psyche*, from the Greek, meaning “soul” or “mind,” a common suffix for delicately winged insects. Gender feminine.

#### Diagnosis.

In the forewing, Sc with three branches. R1 single. MP originates from stem of MP+CuA a little earlier (more basally) than Rs+MA from R. The Rs+MA bifurcation distinctly basad of the first bifurcation of MP. Both fore- and mid basitarsus shorter than remaining four segments in combined length. The basitarsus in hindlegs almost equal to remaining four segments in combined length.

#### Composition.

 Type species only.

#### Comparison.

 In the general venation scheme, the *Jeholopsyche* gen. n. differs from *Aneuretopsyche* Rasnitsyn & Kozlov, 1990 by the Sc in the forewing having three branches, R1 single, and Rs+MA bifurcation distinctly basad of the first forking of MP.

### Jeholopsyche liaoningensis sp. n.

urn:lsid:zoobank.org:act:4CEF36BC-0B9F-4E57-9E00-877367C5E9F1

http://species-id.net/wiki/Jeholopsyche_liaoningensis

Figs 1 [Fig F2] [Fig F3] [Fig F4] [Fig F5] [Fig F6] [Fig F7] [Fig F8] 9

#### Etymology.

 The specific name refers to Liaoning Province, which includes the site of the fossil’s discovery.

#### Holotype.

 An almost complete male specimen with well-preserved body and wings, part and counterpart, specimen numbers CNU-M-LB-2005-002-1 and CNU-M-LB-2005-002-2. Forewing length (preserved part) at least 21.5 mm, width 6 mm; body length (excluding antennae and proboscis) minimally 23 mm; proboscis length 6.8 mm; antenna length (preserved part) minimally 10 mm.

#### Paratype.

A poorly-preserved specimen of unknown sex with body and wings, lateral view, specimen number CNU-M-LB-2005-001 (Ren et al. 2009, Fig. 2G).

#### Locality and horizon.

 Huangbanjigou Village, Shangyuan Township, Beipiao City, Liaoning Province, China. Yixian Formation, of Early Cretaceous (late Barremian) age.

#### Description.

**Figure 1.**
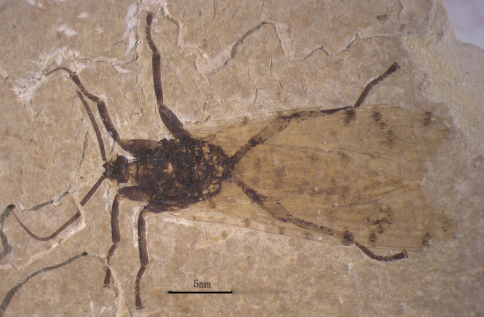
Photo image of *Jeholopsyche liaoningensis*, gen. et sp. n. Holotype, specimen CNU-M-LB-2005-002-1, part.

**Figure 2. F2:**
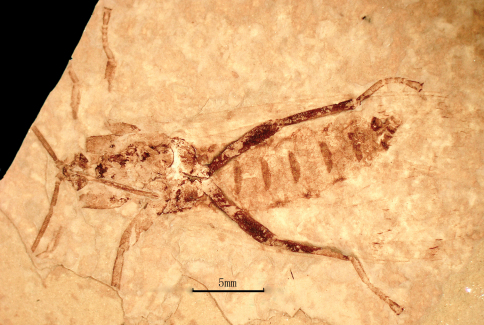
Photo image of *Jeholopsyche liaoningensis* gen. et sp. n. Holotype, specimen CNU-M-LB-2005-002-2, counterpart.

**Figure 3. F3:**
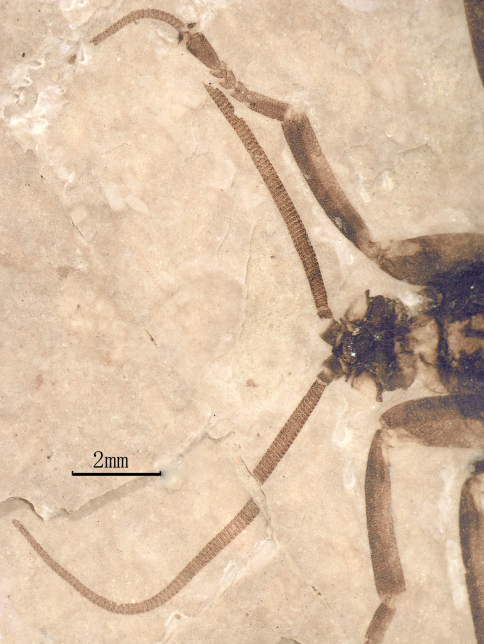
Photo image of head and antennae of *Jeholopsyche liaoningensis* gen. et sp. n. Holotype, specimen CNU-M-LB-2005-002-1.

**Figure 4. F4:**
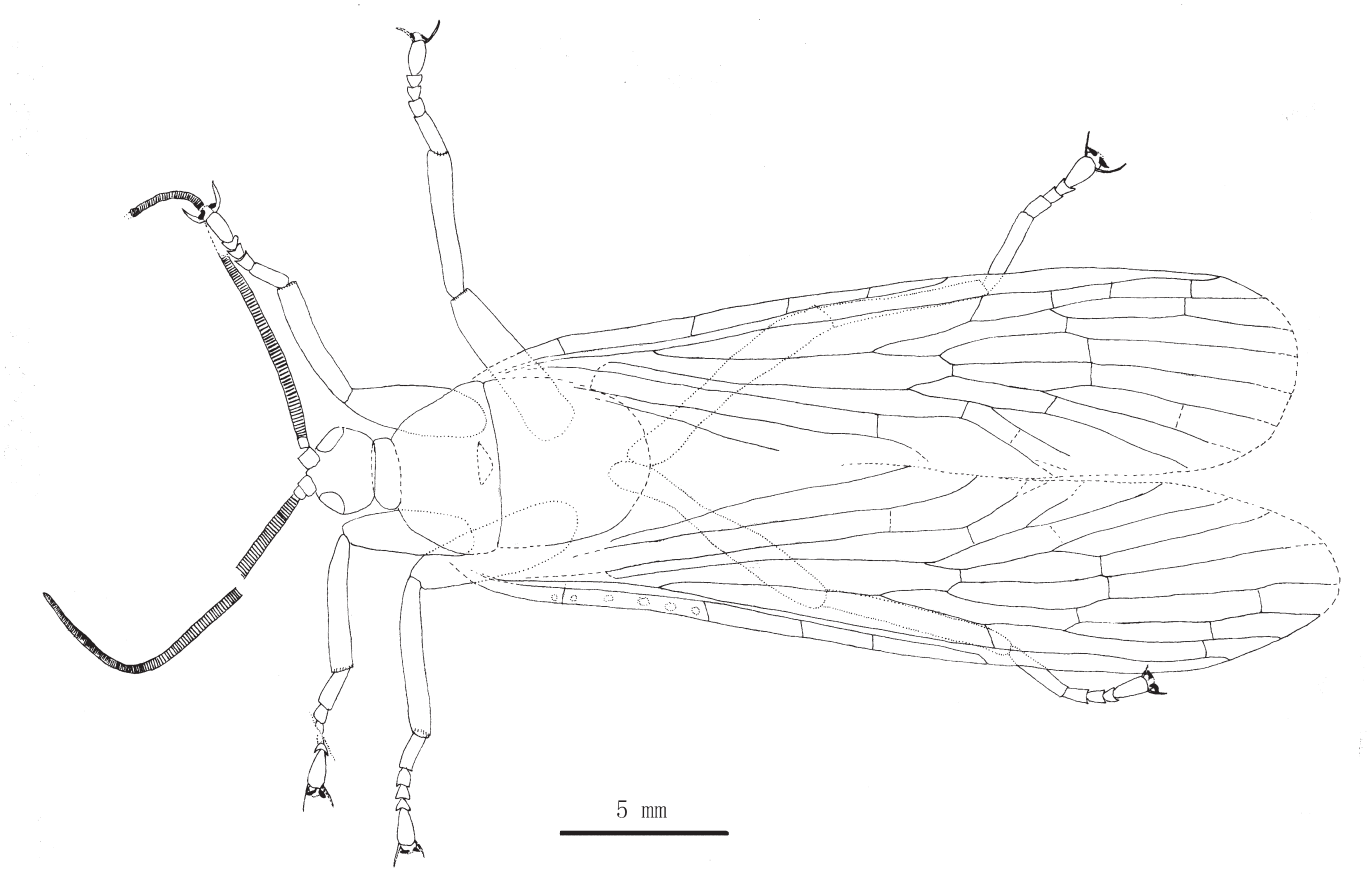
Line drawing of *Jeholopsyche liaoningensis* gen. et sp. n. Holotype in dorsal view, specimen CNU-M-LB2005-002-1.

**Figure 5. F5:**
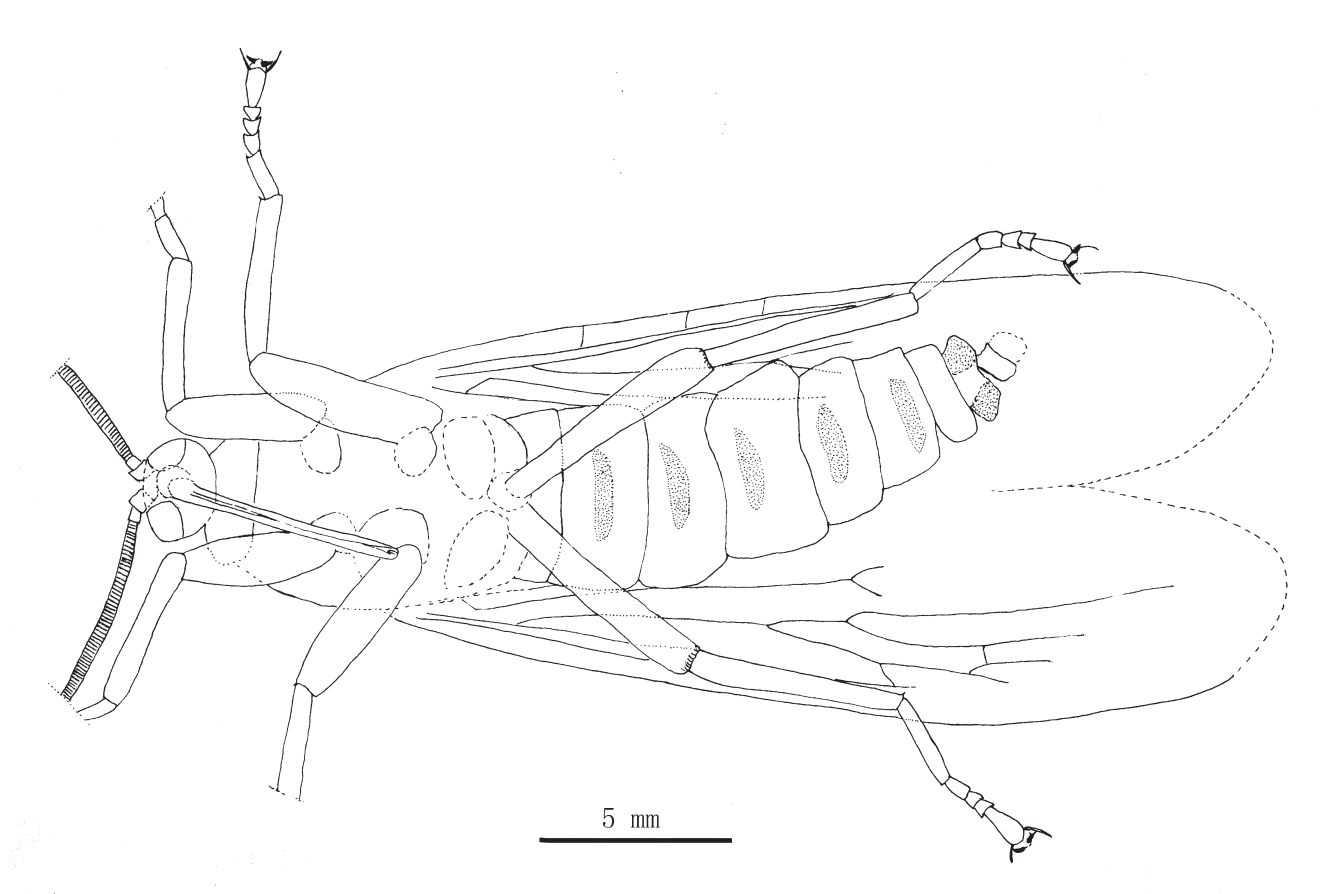
Line drawing of *Jeholopsyche liaoningensis* gen. et sp. n. Holotype in ventral view, specimen CNU-M-LB-2005-002-2.

 Male: The specimen shows details of a nearly complete insect (Figs 1–5). A pair of forewings is almost symmetrically arranged, but hindwings are obscure.

*Head:* Oviform in dorsal view, mouthparts opisthognathous or possibly hypognathous. Eyes large, widely separated. Between eyes, frons and clypeus well-developed. Proboscis ca. 6.8 mm long (proboscis length of paratype, 5.8 mm), straight, composed of indistinct labrum proximally and mostly labium, the latter with a distinct, fleshy pseudolabellum apically; mouth ellipsoidal and subterminally placed (Figs 7–8). Proboscis siphonate, stylets absent; covered with annulate dense hairs or microtrichia. Antennae distinctly longer than proboscis, flagellum multiarticulate, with annulate hairs.

**Figure 6. F6:**
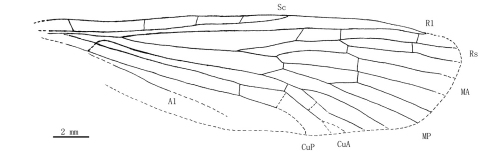
Line drawing of forewing of *Jeholopsyche liaoningensis* gen. et sp. n. Holotype, specimen CNU-M-LB-2005-002-1.

**Figure 7. F7:**
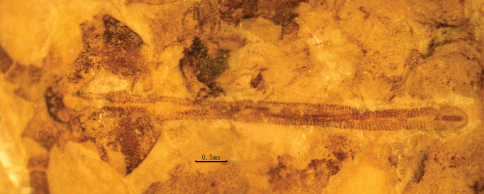
Photo image of proboscis of *Jeholopsyche liaoningensis* gen. et sp. n. Holotype, specimen CNU-M-LB-2005-002-2.

**Figure 8. F8:**
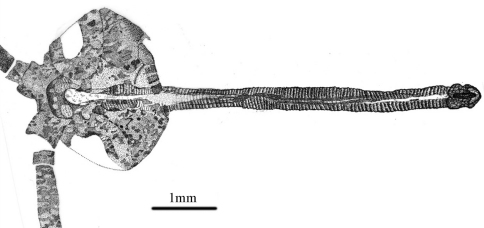
Line drawing of proboscis of *Jeholopsyche liaoningensis* gen. et sp. n. Holotype, specimen CNU-M-LB-2005-002-2.

*Thorax:* Pronotum small; meso- and metanotum more or less similar to each other; both scutum and scutellum not discernible.

**Figure 9.**
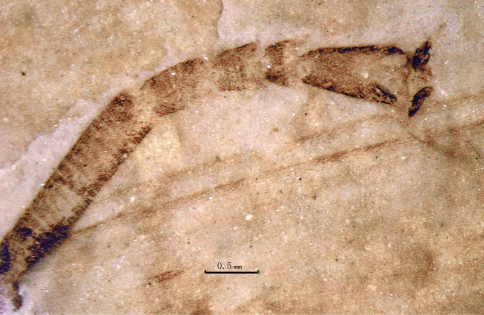
Photo image of hind tarsi of *Jeholopsyche liaoningensis*, gen. et sp. n. Holotype, specimen CNU-M-LB-2005-002-2.

*Legs:* Coxae smaller than those in typical scorpionflies. The legs entirely covered with annulate pubescence. Tarsi 5-segmented. The fore- and midlegs short, almost equal to antennae in length; their basitarsus distinctly shorter than remaining four segments combined in length. The hindlegs somewhat longer and slender, almost equal to forewing in length, with at least 1 apical spur; the basitarsus longest, almost equal to remaining four segments combined in length. All pretarsi with a pair of distinct claws, each developing a reduced and thickened pulvillus (Fig. 9).

*Wings:* Forewing slender (length/width ratio about 3.5:1); apical margin obscure, probably rounded; membrane delicate. A humeral vein present at the base of costal area. Sc long, reaching wing margin almost at same level as the MA bifurcation, with three inclined anterior branches; Sc area with some circular pale spots. Pterostigma probably absent. Both Rs and MA with two endings at or near the apical wing margin; Rs forking later than MA; MP forking later than where MA originates from Rs, with 4 long branches. Thyridium untraceable. Anal area broadened distinctly; A1 well developed. Forewing venational details is depicted in Fig. 6; hindwing untraceable.

*Abdomen:* elongate, tapering apically, with 9 visible segments. Basitergum (T1) small and closely associated with the metathorax; segments 2–6 distinctly longer. Segments 7–8 more slender than 2–6 (Figs 2, 5); segments 9–11 obscure but enlarged, indicating male sex. Cerci unknown.

## Discussion and conclusion

*Jeholopsyche liaoningensis* is the first well-preserved member of the Aneuretopsychidae that exhibits exquisite details of the body, including head and all mouthpart elements, particularly the proboscis. Morphological details of the siphonate proboscis, including setae arranged into annulae, a fleshy pseudolabellum, and an ellipsoidal, subterminal mouth indicate that these insects were fluid feeders on the secretions and exudates from gymnospermous reproductive structures that also co-occur in Yixian deposits. From the proboscis structure and inferred ecological relationships, we conclude that *Jeholopsyche liaoningensis* was a pollinator of gymnosperm hosts that bore deep funnel or other tubular structures laden with nectar-like fluid rewards (Labandeira et al. 2007; Ren et al. 2009). During the mid Early Cretaceous, *Jeholopsyche liaoningensis* also was a member of a diverse guild of long-proboscid insects that included brachycerous files, kalligrammatid lacewings and perhaps early glossate moths (Labandeira 2010).

## Supplementary Material

XML Treatment for Aneuretopsychidae

XML Treatment for Jeholopsyche

XML Treatment for Jeholopsyche liaoningensis

## References

[B1] Bashkuev AS (2011) Nedubroviidae, a new family of Mecoptera: the first Paleozoic long-proboscid scorpionflies. Zootaxa 2895: 47-57.

[B2] Chen W Ji Q Liu D-Y Zhang Y Song B Liu X-Y (2004) Isotope geochronology of the fossil-bearing beds in the Daohugou area, Ningcheng, Inner Mongolia. Geological Bulletin of China 23: 1165-1169.

[B3] Grimaldi DA Engel MS (2005) Evolution of the Insects. New York: Cambridge University Press, 755 pp.

[B4] Grimaldi DA Zhang J-F Fraser NC Rasnitsyn AP (2005) Revision of the bizarre Mesozoic scorpionflies in the Pseudopolycentropodidae (Mecopteroidea). Insect Systematics & Evolution 36: 443-458. 10.1163/187631205794761021

[B5] International Commission on Zoological Nomenclature (ICZN) (1999) International code of zoological nomenclature, 4th edition. International Trust for Zoological Nomenclature, London, 30+306 pp.

[B6] Labandeira CC Kvaček J Mostovski MB (2007) Pollination drops, pollen, and insect pollination of Mesozoic gymnosperms. Taxon 56: 663-695.

[B7] Labandeira CC (2010) The pollination of mid Mesozoic seed plants and the early history of long-proboscid insects. Annals of the Missouri Botanical Garden 97: 469-513. 10.3417/2010037

[B8] Novokshonov VG (2002) Order Panorpida Latreille, 1802. The scorpionflies. In: Rasnitsyn AP Quicke DLJ (Eds). History of Insects. Kluwer Academic Publisher, Dordrecht, Netherlands: 194-198.

[B9] Rasnitsyn AP Kozlov MV (1990) A new group of fossil insects: scorpionfly with cicada and butterfly adaptations. Doklady Akademii Nauk SSSR 310: 973–976. [in Russian]

[B10] Ren D Guo Z-G Lu L-W Ji S-A Tang F Jin Y-G Fang X-S Ji Q (1997) A further contribution to the knowledge of the Upper Jurassic Yixian Formation in western Liaoning. Geological Review 43: 449-460.

[B11] Ren D (1998) Flower-associated Brachycera flies as fossil evidences for Jurassic angiosperm origins. Science 280: 85-88. 10.1126/science.280.5360.85 9525862

[B12] Ren D Labandeira CC Santiago-Blay JA Rasnitsyn AP Shih C-K Bashkuev A Logan MAV Hotton CL Dilcher DL (2009) A probable pollination mode before angiosperms: Eurasian, long-proboscid scorpionflies. Science 326: 840-847. 10.1126/science.1178338 19892981 PMC2944650

[B13] Ren D Labandeira CC Shih C-K (2010a) New Mesozoic Mesopsychidae (Mecoptera) from northeastern China. Acta Geologica Sinica (English Edition) 84: 720-731. 10.1111/j.1755-6724.2010.00244.x

[B14] Ren D Shih C-K Labandeira CC (2010b) New Jurassic pseudopolycentropodids from China (Insecta: Mecoptera). Acta Geologica Sinica 84: 22-30. 10.1111/j.1755-6724.2010.00166.x

[B15] Ren D Shih C-K Gao T-P Yao Y-Z Zhao Y-Y (2010c) Silent Stories - Insect Fossil Treasures from Dinosaur Era of the Northeastern China. Scientific Publishing House, Beijing, 296–310.

[B16] Swisher CC III Wang Y-Q Wang X-L (1999) Cretaceous age for the feathered dinosaurs of Liaoning, China. Nature 400: 58-61. 10.1038/21872

[B17] Swisher CC III Wang X-L Zhou Z-H Wang Y-Q Jin F Zhang J-Y Xu X Wang Y (2002) Further support for a Cretaceous age for the feathered-dinosaur beds of Liaoning, China: New 40Ar/39Ar dating of the Yixian and Tuchengzi Formations. Chinese Science Bulletin (English Version) 47: 135-138.

[B18] Wang S-S Wang Y-Q Hu, H-G Li H-M (2001) The existing time of Sihetun vertebrates in western Liaoning, China. Chinese Science Bulletin (English Version) 46: 779-782. 10.1007/BF03187222

[B19] Wang W-L Zhang L-J Zheng S-L Zheng Y-J Zhang H Li Z-T Young F-L (2004) A new study on the stratotype and biostratigraphy of the Yixian stage in Yixian-Beipiao region, Liaoning - establishment and study of stratotypes of the Yixian stage. Acta Geologica Sinica 78: 433–447. [In Chinese with English abstract]

[B20] Wang W-L Zhang L-J Zheng S-L Ren D Zheng Y-J Ding Q-H Zhang H Li Z-T Yang F-L (2005) The age of the Yixian stage and the boundary of Jurassic-Cretaceous the establishment and study of stratotypes of the Yixian stages. Geological Review 5: 234–242. [In Chinese with English abstract]

[B21] Zheng S-L Zheng Y-J Xing DH (2003) Characeristics, age and climate of Late Jurassic Yixian flora from western Liaoning. Journal of Stratigraphy 27: 233-241.

[B22] Zhou Z Barrett PM Hilton J (2003) An exceptional preserved Lower Cretaceous ecosystem. Nature 421: 807-814. 10.1038/nature01420 12594504

